# Supplementary material for: PTTG1/ZEB1 Axis Regulates E-Cadherin Expression in Human Seminoma
Source: Cancers (Basel). 2022 Oct 5;14(19):4876. doi: 10.3390/cancers14194876 (PMC9564063; doi:10.3390/cancers14194876)
Supplement: Supplementary file 1 [file cancers-14-04876-s001.zip › cancers-1947312-supplementary.pdf]

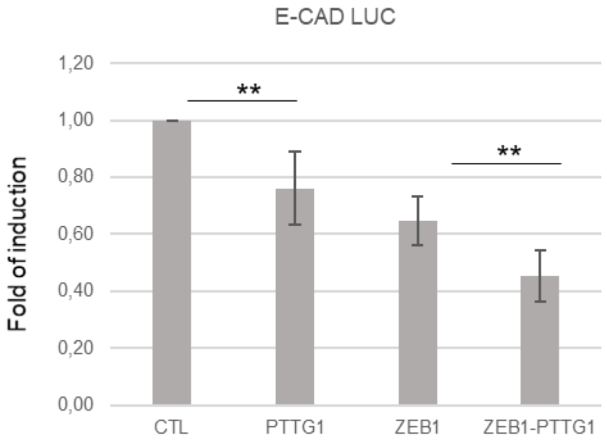

Figure S1. PTTG1 represses E-CAD promoter.
Histogram shows the fold of induction of Luciferase activity in SEM-1 cells transfected with control plasmid (CTL), PTTG1 plasmid (PTTG1), ZEB1 plasmid (ZEB1) and ZEB1 plus PTTG1 (ZEB1+PTTG1), normalized to Renilla signal, set arbitrarily to 1 in control transfection (CTL). Mean ± SD of three independent biological replicates is shown (N = 3; **= $p < 0.05$, two-tailed unpaired t-test).

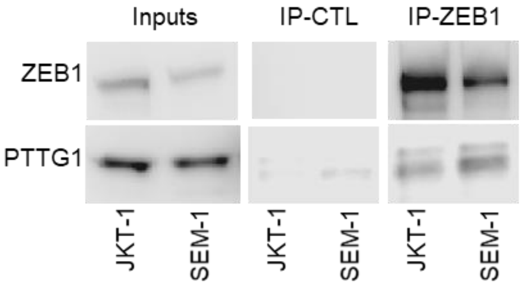

Figure S2. PTTG1 interacts with ZEB1 in seminoma cell lines.
Western blot analysis (Wb) of the indicated proteins. Inputs represents 1/10 of the total protein used in Immunoprecipitation of control (IP-CTL) or ZEB1 (IP-ZEB1) in the indicated cell lines (SEM-1 and JKT-1).

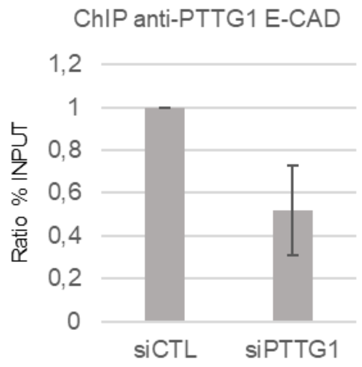

Figure S3. PTTG1 is specifically present on the E-CAD promoter.
Chromatin Immunoprecipitation analysis of PTTG1 protein on E-CAD promoter upon siRNA of PTTG1 (siPTTG1) or control (siCTL) in JKT-1 cells. Histogram shows the ratio of % of input chromatin used for the immunoprecipitation. Control ratio is set arbitrarily to 1.

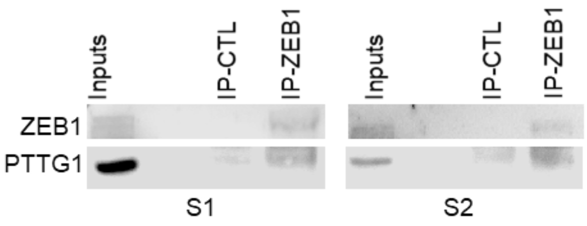

Figure S4. PTTG1 interacts with ZEB1 in vivo in human seminoma specimens.
Western blot analysis (Wb) of the indicated proteins. Inputs represents 1/20 of the total protein used in Immunoprecipitation of control (IP-CTL) or PTTG1 (IP-PTTG1) in the indicated tumor specimens (S1, S2).
